# A novel multimodal model integrating CT-based deep learning and clinical information for predicting 1-year OS of first-line immunotherapy in ES-SCLC patients

**DOI:** 10.3389/fonc.2026.1901931

**Published:** 2026-09-30

**Authors:** Jing Zhang, Liwen Gu, Peixin Dong, Yu Hu, Baomo Liu, Zhihua Wen, Yanbin Zhou, Haohua Yao

**Affiliations:** 1 Department of Pulmonary and Critical Care Medicine, The First Affiliated Hospital of Sun Yat-sen University, Guangzhou, China; 2 Department of Pulmonary and Critical Care Medicine, The Nansha Division of the First Affiliated Hospital of Sun Yat-sen University, Guangzhou, China; 3 Sun Yat-sen University Cancer Center, State Key Laboratory of Oncology in South China; Collaborative Innovation Center for Cancer Medicine, Guangzhou, China; 4 Department of Critical Care Medicine, Sun Yat-sen University Cancer Center, Guangzhou, China; 5 Department of Medical Oncology, State Key Laboratory of Oncology in South China, Collaborative Innovation Center for Cancer Medicine, Sun Yat-sen University Cancer Center, Guangzhou, China; 6 Department of Radiology, The First Affiliated Hospital of Sun Yat-sen University, Guangzhou, China; 7 Department of Urology, Guangdong Provincial People’s Hospital, Guangdong Academy of Medical Sciences, Guangzhou, China

**Keywords:** bone metastasis, deep learning, extensive stage small-cell lung cancer, immunotherapy, pro-gastrin-releasing peptide

## Abstract

**Background:**

Extensive-stage small cell lung cancer (ES-SCLC) is a rare yet highly aggressive malignancy with severely limited treatment options. The efficacy of immunotherapy for ES-SCLC remain limited, with only 14% of patients deriving additional benefit from immune checkpoint inhibitors (ICIs), underscoring significant heterogeneity in patient response. This study aimed to develop a model for the accurate prediction of prognosis and therapeutic response to optimize treatment strategies.

**Methods:**

99 and 25 ES-SCLC patients were enrolled as training and independent internal validation cohorts. Baseline CT images, clinical data, and laboratory markers were collected. Prognostic factors were screened via Cox regression. A mediastinal window CT-based deep learning model (MWDL) was developed and combined with Progastrin-releasing peptide (log(ProGRP + 1)) and bone metastasis to establish the novel MWGB-Cox model.

**Results:**

ProGRP and bone metastasis were independent prognostic factors with area under the curves (AUCs) of 0.705 and 0.666 respectively for predicting 1-year OS. Among the pure deep learning models, the MWDL model achieved better performance with a mean AUC of 0.813 than the deep learning model based on lung window CT images (LWDL, AUC 0.756). Whereas the multimodal MWGB-Cox model not only achieved the highest AUC (0.902) for 1-year OS prediction, but also effectively overcame the limitation of insufficient sensitivity. The predictive performance of the MWGB-Cox model was further validated in the independent internal validation cohort.

**Conclusions:**

This novel, simple, and cost-effective multimodal MWGB-Cox model integrating CT-based deep learning, log(ProGRP + 1), and bone metastasis could robustly predict 1-year OS of ES-SCLC patients receiving first-line immunotherapy, providing a reliable tool for clinical decision-making.

**Significance:**

By integrating CT-based deep learning, log(ProGRP + 1) levels, and bone metastasis, the novel multimodal model achieved the highest predictive AUC (0.902) with improved sensitivity, serving as a simple, cost-effective tool for clinical decision-making.

## Introduction

Small-cell lung cancer (SCLC), characterized by aggressive progression and a high propensity for metastasis, constitutes 10% of all lung cancer cases (1, 2). Owing to its rapid proliferation and early metastatic spread, 70–80% of patients are initially diagnosed with extensive-stage SCLC (ES-SCLC) (3). Although most cases of ES-SCLC are initially sensitive to carboplatin plus etoposide, localized or distant recurrence frequently occurs, resulting in a poor prognosis with median overall survival (OS) of approximately 10 months. A pivotal advancement emerged in 2018 from the IMpower133 trial, which demonstrated that adding atezolizumab (a PD-L1 inhibitor) to etoposide and carboplatin (EC), followed by maintenance atezolizumab, significantly extended median OS to 12.3 months compared to placebo combined with EC (4). This finding was subsequently corroborated by the CASPIAN trial (5), marking a milestone in ES-SCLC therapeutics history. Nevertheless, the addition of immune checkpoint inhibitors (ICIs) yields only a 2 months improvement in 1-year OS (4, 5). Such limited efficacy suggests that clinical benefits may not solely derive from sustained ICI therapy but also reflect heterogeneity in patient responses, indicating the existence of ES-SCLC subgroups that respond to ICIs and others that do not. Consequently, identifying predictive biomarkers or factors influencing ICIs efficacy is critical for optimizing treatment selection, guiding clinical decision-making, and minimizing healthcare costs.

To date, no clinically viable predictors of biomarkers associated with ICIs efficacy in SCLC patients have been established. Although biomarkers such as tumor mutation burden (TMB), tumor PD-L1 expression (6), serum lactate dehydrogenase (LDH), and the GRIm-Score (composed of LDH, albumin, and neutrophil-to-lymphocyte ratio) (7) have demonstrated promise in other malignancies, they have been proved limited predictive utility in SCLC, as evidenced by low area under the curve (AUC) values. Recently, advancements in big data analytics and computational power have opened new avenues for innovation. CT-based artificial intelligence (AI), particularly deep learning (DL), has demonstrated potential in predicting oncological outcomes (8) and may address the limitations of conventional laboratory-based biomarkers (9). For instance, in non-small cell lung cancer (NSCLC), a multimodal model integrating CT-based DL with laboratory data achieved robust predictive performance for ICIs response in advanced NSCLC patients, yielding an AUC of 0.80 (10). Nevertheless, the applicability of CT-based DL combined with laboratory data for predicting ICIs efficacy in ES-SCLC patients remains unknown.

In this study, we evaluated the clinical characteristics and laboratory investigations in ES-SCLC patients, then confirmed Progastrin-releasing peptide (ProGRP) and bone metastases as potential laboratory/clinical biomarkers for further study. Finally, by integrating ProGRP, bone metastases and CT-based DL, we aimed to develop a novel model to predict clinical outcomes in ES-SCLC patients who received immunotherapy as first-line treatment.

## Methods

### Study design and participants

ES-SCLC patients who were treated with first-line etoposide, platinum, and anti- PD-L1 between January 2020 and March 2023 at the First Affiliated Hospital of Sun Yat-sen University (Yuexiu District, Guangzhou, China) were enrolled as the training set. The last follow-up date of the training set was 31 March 2024.

A total of 145 ES-SCLC patients who were treated with first-line etoposide, platinum, and ICIs between January 2020 and March 2023 at the First Affiliated Hospital of Sun Yat-sen University (Yuexiu District, Guangzhou, China) were enrolled. Patients were included if they met the following criteria: (1) were diagnosed as ES-SCLC; (2) were older than 18 years and younger than 80 years; (3) had complete clinical and pathological information; (4) were received etoposide and platinum with anti- PD-L1 treatment as first-line anticancer therapy for at least two courses; (5) with performance status (PS) of 0 or 1. The exclusion criteria were as follows: (1) pregnant or lactating females; (2) history of any cancer; (3) current pulmonary infection before treatment except for obstructive pneumonia caused by ES-SCLC; (4) histological transformation. For instance, patients had an initial diagnosis of NSCLC, with subsequent re-biopsy at the time of disease progression revealing histological transformation to SCLC; (5) the presence of extensive atelectasis or massive pleural effusion at initial diagnosis, which lead to inability to confirm the primary lesion on chest CT for subsequent analysis (6); patients who transferred to other hospitals after two courses of chemoimmunotherapy, including those receiving subsequent-line therapies in other hospitals. Finally, a total of 99 ES-SCLC patients were included in the training set based on pertinent inclusion criteria and exclusion criteria (**Figure 1A**). As of 31 March 2024, the median follow-up was 24.8 months (IQR 16.7–34.5) The median OS of the training set was 16.9 months (95% CI 14.0–29.2).

**Figure 1:**
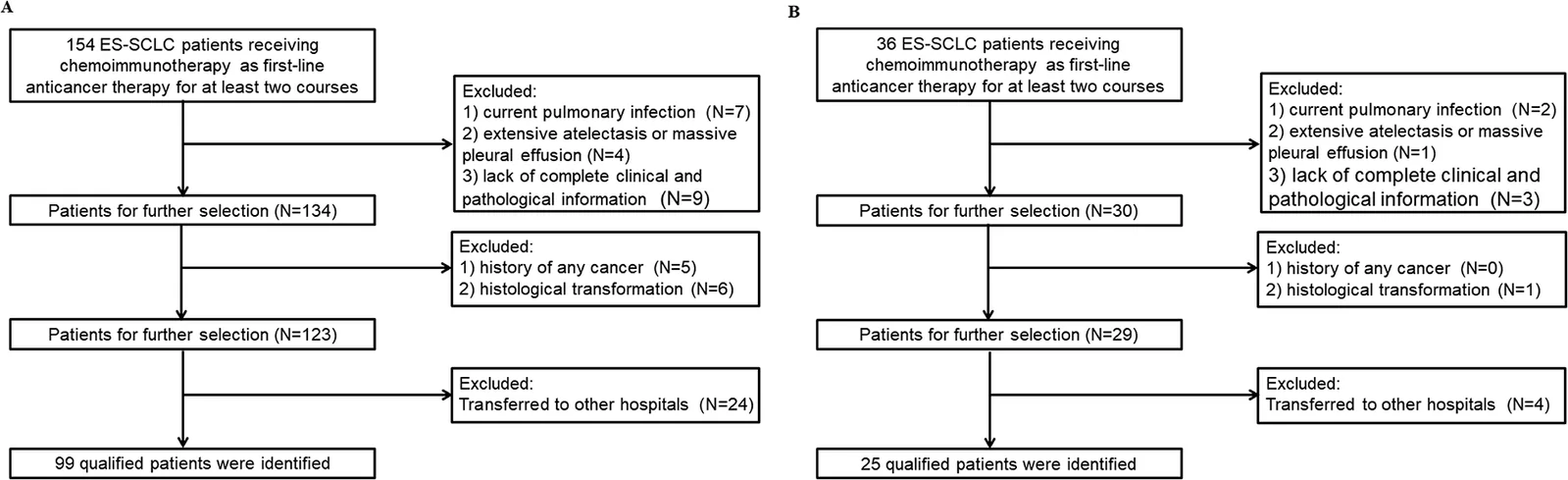
Flow diagram for the exclusion procedure of patientsin the training set **(A)** and in the independent internal validation set **(B)**.

For the selected patients, the staging was determined based on the the 8th lung cancer tumor, node and metastasis (TNM) classification and clinical staging system (11, 12). The histopathologic classification was determined according to the World Health Organization classification (13). Therapeutic response was assessed radiographically after two courses using Response Evaluation Criteria in Solid tumors (RECIST) V.1.1 (14). OS was defined as the time elapsed between the date of treatment initiation and the date of death or the last follow-up date.

### Data collection

Patient clinical data including age, sex, history of drinking, history of smoking, height, weight, body mass index (BMI) (15), cancer metastasis (liver, brain, bone), palliative chest radiotherapy, prophylactic and or palliative brain radiotherapy, blood cell counts, blood biochemistries, and CT scans at baseline were extracted from the hospital’s electronic medical records system. Blood cell counts included white blood cell (16), neutrophil, lymphocyte, monocyte, eosinophil, hemoglobin, and platelet count (17). Blood biochemistries included albumin, LDH, Na (18), Cr (19), NSE and ProGRP. The GRIm-Score is calculated by the following three variables: LDH (within normal range: 0 vs. > upper limit of normal (240 U/L): +1), albumin (≥ 35 g/L: 0 vs. < 35g/L: +1), and neutrophil-to-lymphocyte ratio (NLR) (≤ 6: 0 vs. > 6: +1) (16).

### Prediction efficiency based on clinical features and biomarkers

To investigate factors related to treatment response and prognosis, both univariate Cox regression and multivariate Cox regression were utilized. A multivariate analysis was conducted on variables with a p-value less than 0.05 from the univariate analyses. A meta-analysis by Zhang et al. that included 77 phase III randomised controlled trials across 15 tumor types and 52,342 patients demonstrated that 1-year OS correlated more strongly with patient clinical outcomes than objective response rate (ORR), disease control rate (DCR), or progression free survival (PFS) (20). These associations remained robust across predefined subgroups stratified by tumor type, blinding method, line of therapy, drug target, treatment strategy, and follow up duration. Thus Zhang et al. concluded that 1-year milestone survival was the only validated surrogate endpoint for immunotherapy outcomes. Additionally, pivotal phase III trials in IMpower133 (4) and CASPIAN (5), have used the 1-year OS rate difference between treatment arms as a primary efficacy indicator. Accordingly, we classified patients into OS ≥ 1 year and OS <1 groups for subsequent predictive modelling. A receiver operating characteristic (ROC) curve was used to evaluate the predictive value of clinical features and biomarkers. Assessment indices included AUC, sensitivity and specificity. Statistical analysis was conducted at a 0.05 significance level.

### CT image acquisition and pre-processing

All patients underwent CT screening using a multi-slice spiral CT scanner (Aquilion One 320, Toshiba) before treatment. The CT slices were downloaded in Digital Imaging and Communications in Medicine (DICOM) format and then converted to Joint Photographic Experts Group (JPG) images at the window of lung fields and mediastinum fields.

As shown in **Figure 2A**, CT images of the first quartile (Q1), second quartile (Q2), and third quartile (Q3) of primary SCLC lesions were selected, including lung and mediastinal windows. The region of interest (ROI) of each CT image, containing the tumor and surrounding tissue, was cropped using a rectangular box by two experienced chest radiologists. A third radiologist only participated when there was disagreement over the ROI.

**Figure 2:**
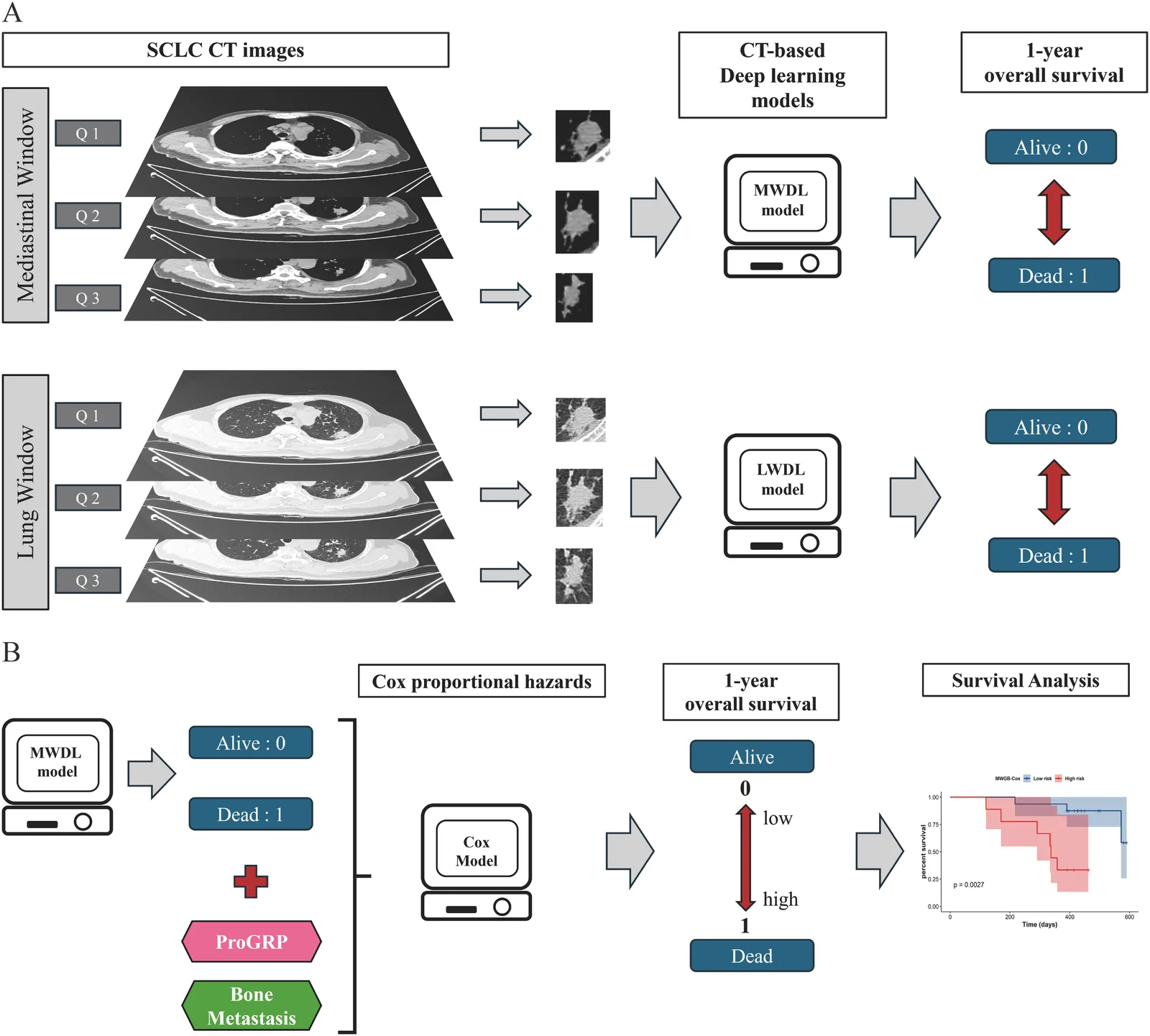
Overview of the deep learning model processing CT images of ES-SCLC primary lesions **(A)** and the construction of novel MWGB-Cox model **(B)**. **(A)** Representative baseline CT images of the primary tumor at three axial levels (Q1, superior; Q2, middle; Q3, inferior). The upper row shows images under the lung window (width, 1500 HU; level, -600 HU), and the lower row shows the corresponding images under the mediastinal window (width, 350 HU; level, 40 HU). Rectangular boxes indicate the regions of interest (ROIs) cropped for deep learning analysis. These three slices were stacked as three input channels of the ResNet-34 network (Channel 1 = Q1, Channel 2 = Q2, Channel 3 = Q3) to form a pseudo-RGB tensor. **(B)** Schematic of the Cox proportional hazards model incorporating MWDL predictions, ProGRP (log(ProGRP + 1)), and bone metastasis to generate the final MWGB-Cox model for predicting 1-year OS.

### CT-based Deep learning algorithm

An end-to-end residual neural network is used to predict a patient’s one-year survival status based on tumor CT images. The architecture of the network is ResNet-34 (21), a 2D architecture which has an input layer of three channels. As depicted in **Figure 2A**, the three representative axial CT slices selected from the superior (Q1), middle (Q2), and inferior (Q3) portions of the tumor are stacked along the channel dimension to form a pseudo-RGB tensor. Accordingly, Channel 1 receives the Q1 slice, Channel 2 receives the Q2 slice, and Channel 3 receives the Q3 slice. This approach enables the convolutional kernels to simultaneously extract and synthesize cross-sectional imaging features across the entire tumor volume. Furthermore, to avoid overfitting due to the relatively small sample size, we embedded a dropout layer between the linear layer and the SoftMax function.

During the model development, five-fold cross-validation approach that involved randomly splitting the full set of our CT image dataset into five balanced subsets was applied to train and confirm the performance of deep learning models. All patients were split into five partitions before training, keeping the ratio of OS ≥ 1 year and OS <1 year balanced between partitions. In each fold of cross-validation, four portions were used to fit the model, and the remaining portion was used to validate the model. These out-of-fold predictions were compiled to form the complete patient-level DL prediction vector, which was then used to train the Cox proportional hazards model, ensuring no data leakage between deep learning model training and survival model construction.

Models were evaluated at the patient level using the AUC, sensitivity, and specificity in each fold of validation. The average AUC of all five folds represented the overall performance of the models. All deep learning experiments were implemented using Python under the PyTorch framework and trained on NVIDIA GeForce RTX 2080Ti GPUs.

### Cox proportional hazards model based on deep learning predictions, clinical features and biomarkers

Patient-level deep learning predictions (DL predictions) for the training dataset were obtained from the CT-based DL model in each fold validation set in cross-validation. Then, a Cox proportional hazards model (Cox model) was constructed using DL predictions, biomarkers or clinical features to predict the response of immunotherapy in ES-SCLC patients by outputting the probability of 1-year OS status (**Figure 2B**). The Cox model was evaluated using five-fold cross-validation, with the same patient groups being retained as in the development phase of the CT-based DL model.

### Independent internal validation

Another independent set of ES-SCLC patients who were treated with first-line etoposide, platinum, and ICIs between April 2023 to April 2024 at the Nansha Division of the First Affiliated Hospital of Sun Yat-sen University (Nansha District, Guangzhou, China) were included as the independent internal validation set. The last follow-up date of the independent internal validation set was 31 July 2026.

A total of 33 ES-SCLC patients who were treated with first-line etoposide, platinum, and ICIs between April 2023 to April 2024 at the Nansha Division of the First Affiliated Hospital of Sun Yat-sen University (Nansha District, Guangzhou, China) were enrolled. As shown in **Figure 1B**, 25 cases of qualified ES-SCLC patients were identified as the independent internal validation set for further analysis. As of 31 July 2026, the median follow-up was 27.9 months (IQR 27.5–31.3). The median OS of the independent internal validation set was 16.1 months (95% CI 11.3–28.4).

All the demographic and clinical characteristics are shown in **Table 1**. All included patients had complete data for the variables listed in **Table 1**, with zero missing values for clinical, laboratory, and imaging variables in both cohorts. Of the 99 ES-SCLC patients enrolled in the training set, 70 patients (71%) reached PR, 17 patients (17%) reached SD and 12 patients (12%) reached PD after two courses treatment. The objective response rate was 71%, whereas the disease control rate was 88%. Of the 25 ES-SCLC patients enrolled in the independent internal validation set, 16 patients (64%) reached PR, 5 patients (20%) reached SD and 4 patients (16%) reached PD after two courses treatment. The objective response rate was 64%, whereas the disease control rate was 84%. The patients with OS ≥ 1 year had a significantly much higher proportion of radiotherapy than patients with OS < 1 year in both the training set and the independent internal validation set.

**Table 1:** Clinical characteristics of all study participants.

Clinical	Training set (N = 99)		Validation set (N = 25)		P value
	OS≥1 year	OS<1 year	OS≥1 year	OS<1 year	
Gender					
Male	71 (92%)	21 (95%)	17 (95%)	7 (100%)	0.94
Female	6 (8%)	1 (5%)	1 (5%)	0 (0%)	
Age	60.6 ± 1.0	61.6 ± 2.0	62.3 ± 2.1	64.6 ± 2.9	0.12
Smoke					
Yes	56 (73%)	21 (95%)	15 (83%)	5 (71%)	0.13
No	21 (27%)	1 (5%)	3 (17%)	2 (29%)	
Alcohol					
Yes	31 (40%)	7 (32%)	7 (39%)	2 (29%)	0.82
No	46 (60%)	15 (68%)	11 (61%)	5 (71%)	
Primary lesion					
Central	63 (82%)	18 (82%)	15 (83%)	6 (86%)	0.96
Peripheral	14 (18%)	4 (18%)	3 (17%)	1 (14%)	
Liver metastases					
Yes	18 (23%)	11 (50%)	3 (17%)	2 (29%)	0.45
No	59 (77%)	11 (50%)	15 (83%)	5 (71%)	
Brain metastases					
Yes	14 (18%)	5 (23%)	2 (11%)	2 (29%)	0.98
No	63 (82%)	17 (77%)	16 (89%)	5 (71%)	
Bone metastases					
Yes	20 (26%)	13 (59%)	3 (17%)	2 (29%)	0.23
No	57 (74%)	9 (41%)	15 (83%)	5 (71%)	
BMI (kg/m²)	22.5 ± 0.3	21.3 ± 0.5	23.3 ± 0.7	23.3 ± 1.1	0.73
WBC (*10^9^)	8.2 ± 0.4	8.4 ± 0.7	8.7 ± 0.8	8.1 ± 1.0	0.92
NEUT (*10^9^)	5.7 ± 0.4	6.0 ± 0.6	6.4 ± 0.7	5.4 ± 0.9	0.83
LY (*10^9^)	1.3 ± 0.1	1.5 ± 0.2	1.4 ± 0.2	1.8 ± 0.2	0.21
MO (*10^9^)	0.8 ± 0.1	0.7 ± 0.1	0.7 ± 0.1	0.5 ± 0.1	0.11
EO (*10^9^)	0.2 ± 0.0	0.1 ± 0.0	0.1 ± 0.0	0.3 ± 0.1	0.09
Hb (g/L)	133.8 ± 1.6	128.9 ± 4.3	133.7 ± 2.6	131.1 ± 4.8	0.56
PLT (*10^9^)	295.5 ± 12.0	305.1 ± 21.4	289.3 ± 18.6	314.0 ± 36.3	0.93
Na (mmol/L)	136.8 ± 0.7	137.1 ± 1.0	134.1 ± 2.3	138.4 ± 1.0	0.08
Cr (umol/L)	76.1 ± 2.2	71.2 ± 3.3	72.0 ± 3.8	78.4 ± 10.4	0.10
Brain radiotherapy	32 (42%)	5 (23%)	7 (38%)	1 (14%)	0.71
Chest radiotherapy	26 (34%)	1 (5%)	6 (33%)	0 (0%)	0.95
Biomarkers					
GRIM-Score	0.9 ± 0.1	1.4 ± 0.2	0.9 ± 0.2	1.6 ± 0.3	0.88
NSE (ng/ml)	79.2 ± 9.3	210.6 ± 80.0	111.6 ± 24.6	148.3 ± 44	0.74
ProGRP (pg/ml)	1995.0 ± 482.0	9094.0 ± 3076.0	2341.0 ± 722.6	4752.0 ± 3047.0	0.75

Patients in the independent internal validation set were collected from the Nansha Division of the First Affiliated Hospital of Sun Yat-sen University (Nansha District, Guangzhou, China) to further evaluate the CT-based DL model and Cox model. Keeping the training parameters constant with those in the five-fold cross-validation, we re-trained the DL model using CT images of all patients in the training set, and predicted each patient in the independent internal validation set. In the same way, we re-trained the Cox model using all the patients in the training set and evaluated it on the independent internal validation set.

### Statistical analysis

The primary outcome was the accuracy of the novel models in predicting the 1-year OS. R version 3.5 (The R Foundation for Statistical Computing) and MedCalc Statistics were used for all analyses. The area under the ROC curve (AUC) was calculated to evaluate the predictive value. Results were expressed as hazard ratios (HRs) and 95% confidence intervals (CIs). AUC of ≥0.7 was used to denote a good predictive value, and a two-sided p<0.05 was considered significant. For the independent validation cohort, time-dependent ROC curves for 1-year overall survival were constructed using the inverse probability of censoring weighting (IPCW) estimator (weighting = ‘marginal’) via the time ROC R package, which appropriately accounts for censored observations. For the 5-fold cross-validation, binary ROC analysis was employed using the 1-year survival status as the gold standard, because the small validation fold size rendered IPCW-based time-dependent ROC estimation unstable. The proportional hazards assumption for the final Cox model was formally tested using Schoenfeld residual tests (survival R package). Schoenfeld residual plots are provided in **Supplementary Figure S2**. Nonlinearity of continuous variables was assessed using restricted cubic splines (4 knots) via the rms R package. Calibration was assessed using bootstrap-corrected calibration curves (B = 300) via the calibrate function in the rms R package. The integrated Brier score (IBS) was calculated using the pec R package, with the null Kaplan-Meier model as the reference.

## Results

### Prediction efficiency based on clinical features and biomarkers

To identify independent predicting factors for patients receiving ICIs, we employed both univariate and multivariate Cox regression analyses (**Table 2**) in the training set. In the univariate Cox regression, liver metastases (p < 0.01), bone metastases (p < 0.01), chest radiotherapy (p < 0.01), GRIM-Score (p < 0.01), NSE (p < 0.01) and ProGRP (p < 0.01) were significant factors. However, in the following multivariable Cox regression analyses, liver metastases (HR = 1.52; 95% CI = 0.81-2.86; p = 0.20), chest radiotherapy (HR = 0.64; 95% CI = 0.29-1.43; p = 0.28), GRIM-Score (HR = 1.40; 95% CI = 1.00-1.97; p = 0.05) and NSE (HR = 1.00; 95% CI = 1.00-1.00; p = 0.34) did not achieve statistical significance, whereas ProGRP (HR = 1.00; 95% CI = 1.00-1.00; p < 0.01) and bone metastases (HR = 2.77; 95% CI = 1.51-5.09; p < 0.01) were proved to be statistically significant prognostic biomarkers.

**Table 2:** Prognostic factors for OS of all study participants.

Characteristics	Univariable analysis			Multivariable analysis		
	HR	95% CI	P value	HR	95% CI	P value
Gender	1.237	0.494-3.099	0.649	N/A	N/A	N/A
Age	1.014	0.982-1.046	0.396	N/A	N/A	N/A
Smoke	1.450	0.765-2.747	0.255	N/A	N/A	N/A
Alcohol	0.941	0.554-1.601	0.823	N/A	N/A	N/A
Primary lesion	0.954	0.544-1.674	0.869	N/A	N/A	N/A
Liver metastases	2.767	1.569-4.881	**<0.001**	1.517	0.805-2.856	0.197
Brain metastases	1.521	0.809-2.861	0.209	N/A	N/A	N/A
Bone metastases	3.107	1.783-5.414	**<0.001**	2.768	1.505-5.090	**0.001**
BMI	0.942	0.855-1.039	0.229	N/A	N/A	N/A
WBC	1.038	0.963-1.118	0.335	N/A	N/A	N/A
NEUT	1.041	0.966-1.122	0.293	N/A	N/A	N/A
LY	1.159	0.801-1.678	0.434	N/A	N/A	N/A
MO	0.703	0.391-1.261	0.237	N/A	N/A	N/A
EO	0.725	0.173-3.041	0.660	N/A	N/A	N/A
Hb	0.996	0.977-1.014	0.644	N/A	N/A	N/A
PLT	1.001	0.999-1.003	0.499	N/A	N/A	N/A
Na	1.005	0.955-1.056	0.859	N/A	N/A	N/A
Cr	0.999	0.983-1.015	0.895	N/A	N/A	N/A
Brain radiotherapy	0.774	0.432-1.387	0.383	N/A	N/A	N/A
Chest radiotherapy	0.372	0.180-0.768	**0.003**	0.641	0.287-1.429	0.277
GRIM-Score	1.564	1.150-2.126	**0.004**	1.403	0.998-1.970	0.051
NSE	1.002	1.000-1.003	**0.001**	0.999	0.997-1.008	0.343
ProGRP	1.000	1.000-1.000	**<0.001**	1.000	1.000-1.000	**0.001**

Bold and underlined values indicate statistically significant differences (P < 0.05).

Then, the levels of ProGRP and the proportion of bone metastasis were compared between OS ≥ 1 year patients group and OS < 1 year patients group. As shown in **Figures 3A, B**, patients with OS ≥ 1 year have a lower levels/proportion of ProGRP and bone metastasis than those with OS < 1 year. To determine the diagnostic values of the ProGRP and bone metastasis, we performed ROC analysis to evaluate the capability of ProGRP and bone metastasis in predicting 1-year OS. As shown in **Figure 3C**, the AUCs of ProGRP and bone metastasis were 0.705 and 0.666 respectively, while the AUC of ProGRP plus bone metastasis was 0.721.

**Figure 3:**
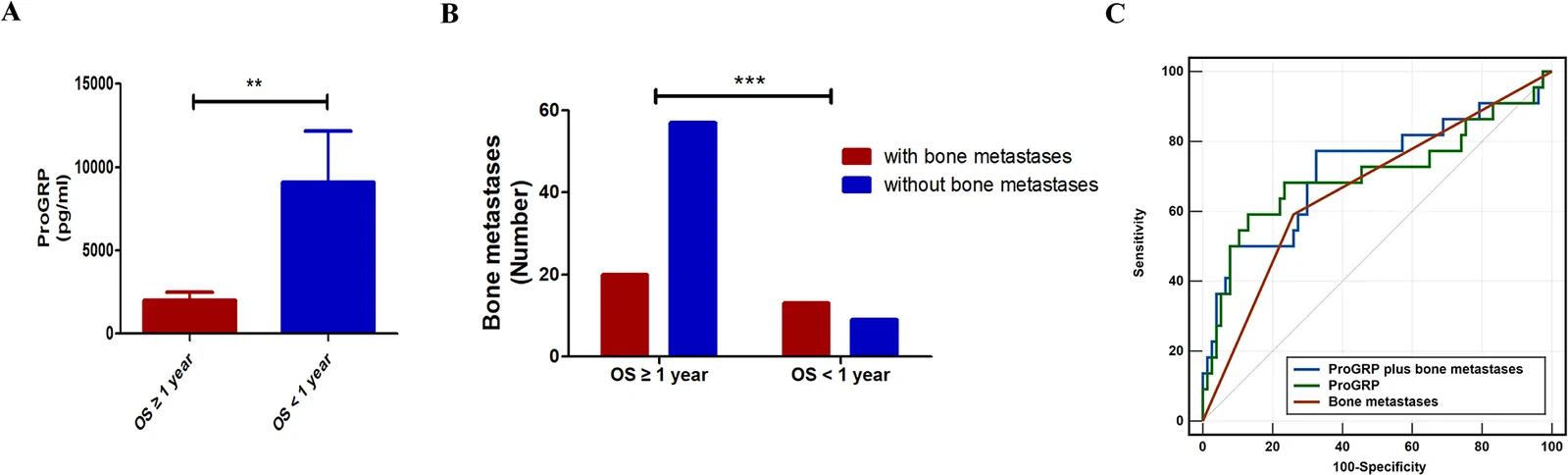
Comparison of the levels/proportion and the prediction efficiency between ProGRP and bone metastasis. **(A)** Comparison of the ProGRP levels between OS ≥ 1 year patients group and OS < 1 year patients group. **(B)** Comparison of the bone metastasis numbers between OS ≥ 1 year patients group and OS < 1 year patients group. **(C)** Compare the AUCs of ProGRP, bone metastasis, and their combination for predicting 1-year OS in ES-SCLC patients who received immunotherapy as first-line treatment. **P < 0.01, ***P < 0.001.

### Performance between deep learning models and Cox proportional hazards models in cross-validation

This study evaluated the patient-level predictive performance of five models using five-fold cross-validation (**Table 3**). Representative baseline CT images of the primary tumor under the lung window (LW) and mediastinal window (MW) settings are shown in **Figure 2A**. Among the pure deep learning models, the deep learning model based on mediastinal window CT images (MWDL) achieved the best performance with a mean AUC of 0.813 ± 0.045, significantly superior to the deep learning model based on lung window CT images (LWDL, mean AUC 0.756 ± 0.053). While maintaining comparable sensitivity (0.690 ± 0.082), MWDL exhibited higher specificity (0.937 ± 0.077 vs. 0.822 ± 0.125), suggesting that mediastinal window CT imaging features carry more important prognostic value for lung cancer outcome prediction.

**Table 3:** The performance of deep learning models and Cox proportional hazards models at each fold in the fivefold cross-validation.

	Patient-level metrics ( 95% CI )										
	LWDL^1^			MWDL^2^			MWGRP-Cox^3^				
	AUC	SEN	SPE	AUC	SEN	SPE	AUC	SEN			SPE
Fold 1	0.719 (0.476-0.894)	0.750 (0.194-0.994)	0.688 (0.413-0.890)	0.844 (0.614-0.965)	0.750 (0.194 – 0.994)	0.938 (0.698-0.998)	0.969 (0.845-1.000)	1.000 (0.398 – 1.000)			0.875 (0.617-0.984)
Fold 2	0.719 (0.476-0.894)	0.500 (0.068-0.932)	0.938 (0.698-0.998)	0.875 (0.651-0.979)	0.750 (0.194-0.994)	1.000 (0.794-1.000)	0.984 (0.896-1.000)	1.000 (0.398-1.000)			0.938 (0.698-0.998)
Fold 3	0.844 (0.614-0.965)	1.000 (0.398-1.000)	0.688 (0.413-0.890)	0.781 (0.543-0.932)	0.750 (0.194-0.994)	0.813 (0.544-0.960)	0.750 (0.449-1.000)	0.750 (0.194-0.994)			0.812 (0.544-0.960)
Fold 4	0.733 (0.491- 0.903)	0.600 (0.147-0. 947)	0.867 (0.595-0.983)	0.767 (0.527-0.924)	0.600 (0.147-0.947)	0.933 (0.681-0.998)	0.773 (0.508-1.000)	0.800 (0.284-0.995)			0.867 (0.595-0.983)
Fold 5	0.764 (0.517-0.925)	0.600 (0.147-0. 947)	0.929 (0.661-0.998)	0.800 (0.556-0.945)	0.600 (0.147-0. 947)	1.000 (0.768-1.000)	0.914 (0.734–1.000)	0.800 (0.284-0.995)			0.929 (0.661-0.998)
Average*	**0.756 ± 0.053**	0.690 ± 0.195	0.822 ± 0.125	**0.813 ± 0.045**	0.690 ± 0.082	0.937 ± 0.077	**0.878 ± 0.110**	0.870 ± 0.120			0.884 ± 0.051

	Patient-level metrics ( 95% CI )										
	MWB-Cox^4^			MWGB-Cox^5^			/				
	AUC	SEN	SPE	AUC	SEN	SPE	/		/	/	
Fold 1	0.977 (0.922-1.000)	1.000 (0.398-1.000)	0.938 (0.698-0.998)	0.984 (0.941-1.000)	1.000 (0.398–1.000)	0.938 (0.698-0.998)	/		/	/	
Fold 2	0.836 (0.610-1.000)	1.000 (0.398-1.000)	0.563 (0.299-0.802)	1.000 (1.000-1.000)	1.000 (0.398-1.000)	1.000 (0.794-1.000)	/		/	/	
Fold 3	0.852 (0.681-1.000)	1.000 (0.398-1.000)	0.563 (0.299-0.802)	0.766 (0.471-1.000)	0.500 (0.068-0.932)	0.938 (0.698-0.998)	/		/	/	
Fold 4	0.833 (0.589- 1.000)	0.800 (0.284-0.995)	0.733 (0.449-0.922)	0.773 (0.508-1.000)	0.800 (0.284-0.995)	0.867 (0.595-0.983)	/		/	/	
Fold 5	0.957 (0.889-1.000)	1.000 (0.478-1.000)	0.786 (0.492-0.953)	0.986 (0.910-1.000)	1.000 (0.478-1.000)	0.929 (0.661-0.998)	/		/	/	
Average*	**0.891 ± 0.070**	0.960 ± 0.089	0.716 ± 0.159	**0.902 ± 0.121**	0.860 ± 0.219	0.934 ± 0.047	/		/	/	

^1^ LWDL: Deep Learning model based on Lung Window CT images.

^2^ MWDL: Deep Learning model based on Mediastinal Window CT images.

^3^ MWGRP-Cox: Cox proportional hazards model based on MWDL prediction and log(ProGRP+1).

^4^ MWB-Cox: Cox proportional hazards model based on MWDL prediction and bone metastasis.

^5^ MWGB-Cox: Cox proportional hazards model based on MWDL prediction, log(ProGRP+1) and bone metastasis.

^*^ Mean ± Standard Deviation.

Bootstrap variable selection stability (B = 500) confirmed high stability for all three variables: MWDL prediction (95.4%), log(ProGRP) (89.8%), and bone metastasis (94.2%) (**Supplementary Figure S5**).

Bold and underlined values indicate the mean AUC across five folds.

Having confirmed the superiority of the MWDL model, we developed three Cox proportional hazards models incorporating MWDL predictions with ProGRP and/or bone metastasis. In the initial multivariable Cox analysis for variable selection, ProGRP was entered as a continuous variable. Based on subsequent restricted cubic spline analysis demonstrating significant nonlinearity (nonlinear component p = 0.012; **Supplementary Figure S3**), the final MWGRP-Cox and MWGB-Cox models used log(ProGRP + 1), which improved model fit (AIC 334.8 vs. 339.7) and calibration (bootstrap-corrected slope 0.930 vs. 0.833). The MWGRP-Cox model, combining MWDL predictions with log(ProGRP + 1) alone, achieved a mean AUC of 0.878 ± 0. 110, substantially higher than MWDL (0.813 ± 0.045), demonstrating the additive value of this biomarker. The MWB-Cox model, combining MWDL predictions with bone metastasis alone, achieved a mean AUC of 0.891 ± 0.070 with remarkably high sensitivity of 0.960 ± 0.089. While the MWGB-Cox model, integrating MWDL prediction, log(ProGRP + 1) and bone metastasis, achieved the highest AUC value of 0.902 ± 0.121, and maintained favorable sensitivity (0.860 ± 0.219) and specificity (0.934 ± 0.047).

### Performance in the independent internal validation set

The ROC curves and confusion matrices of six different methods in the independent internal validation set are shown in **Figure 4**, and more detailed evaluation metrics are presented in **Table 4**. Consistent with findings in the training cohort, bone metastasis and ProGRP also demonstrated limited predictive performance in the validation cohort, with AUC values of 0.730 (95% CI: 0.519–0.942) and 0.690 (95% CI: 0.475–0.906), respectively. The MWDL improved predictive accuracy, yielding an AUC of 0.774 (95% CI: 0.573–0.975). The MWGRP-Cox model, integrating MWDL and log(ProGRP + 1), further enhanced predictive performance with an AUC of 0.817 (95% CI: 0.609–1.000), whereas the MWB-Cox model combining MWDL and bone metastasis achieved a higher AUC of 0.853 (95% CI: 0.671–1.000). Moreover, the MWGRP-Cox and MWB-Cox models incorporating log(ProGRP + 1) or bone metastasis, respectively, showed markedly improved sensitivity compared with the MWDL model. The MWGB-Cox model, which integrated MWDL, log(ProGRP + 1), and bone metastasis, exhibited optimal overall performance, with an AUC of 0.873 (95% CI: 0.693–1.000), a sensitivity of 0.857 (95% CI: 0.421–0.996), and a specificity of 0.833 (95% CI: 0.586–0.964). This model also achieved the highest F1-score (0.750) and C-index (0.796), indicating superior overall classification balance. The MWGB-Cox model demonstrated robust generalizability across patient subgroups, with consistent discriminative performance by bone metastasis status and ProGRP tertiles (**Supplementary Table S2**), and no significant interaction between variables (p = 0.163). In addition, calibration curves, predicted probability distributions, and stability analyses of the MWGB-Cox model were produced based on training cohort. Details are provided in **Supplementary Figures S1, S4, S5** and **Supplementary Table S1**.

**Figure 4:**
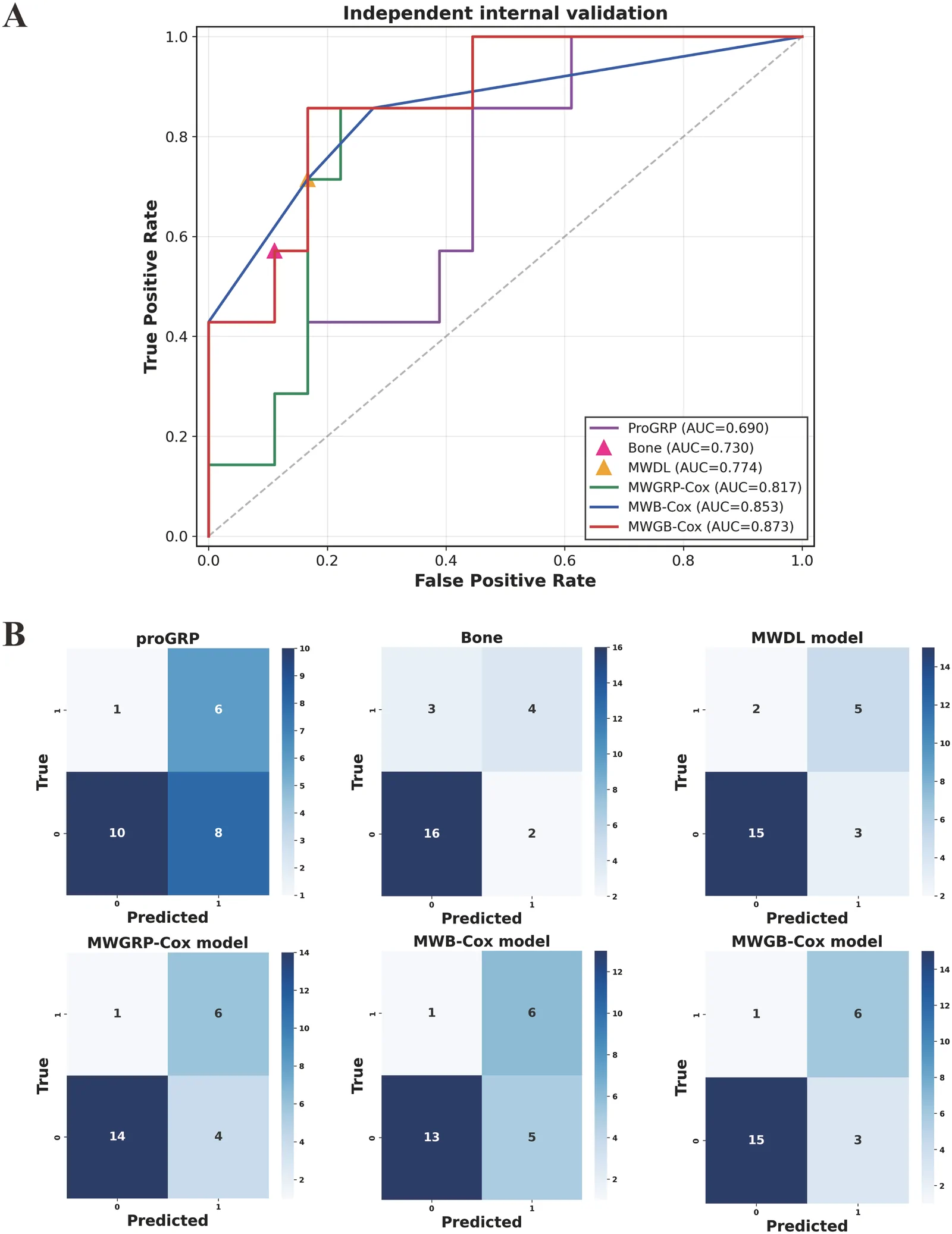
Performance of different methods in the independent internal validation set. **(A)** Receiver operating characteristic (ROC) curves comparing six predictive methods for 1-year overall survival: ProGRP, bone metastasis (Bone), mediastinal window deep learning (MWDL) model, MWGRP-Cox (MWDL and log(ProGRP + 1)), MWB-Cox (MWDL and bone metastasis), and MWGB-Cox (MWDL, log(ProGRP + 1) and bone metastasis). **(B)** Confusion matrices depicting the classification performance of each method.

**Table 4:** Performance of different methods in the independent internal validation.

Methods	AUC (95% CI)	Sensitivity (95% CI)	Specificity (95% CI)	F1 score	C-index
ProGRP	0.690 (0.475-0.906)	0.857 (0.421-0.996)	0.556 (0.308-0.785)	0.571	–
Bone metastasis	0.730 (0.519-0.942)	0.571 (0.184-0.901)	0.889 (0.653-0.986)	0.615	–
MWDL^1^	0.774 (0.573-0.975)	0.714 (0.290-0.963)	0.833 (0.586-0.964)	0.667	–
MWGRP-Cox^2^	0.817 (0.609-1.000)	0.857 (0.421-0.996)	0.778 (0.524-0.936)	0.706	0.741
MWB-Cox^3^	0.853 (0.671-1.000)	0.857 (0.421-0.996)	0.722 (0.465-0.903)	0.667	0.765
**MWGB-Cox** ^4^	**0.873 (0.693-1.000)**	**0.857 (0.421-0.996)**	**0.833 (0.586-0.964)**	**0.750**	**0.796**

^1^ MWDL: Deep Learning model based on Mediastinal Window CT images.

^2^ MWGRP-Cox: Cox proportional hazards model based on MWDL prediction and log (ProGRP+1).

^3^ MWB-Cox: Cox proportional hazards model based on MWDL prediction and bone metastasis.

^4^ MWGB-Cox: Cox proportional hazards model based on MWDL prediction, log (ProGRP+1) and bone metastasis.

Bold values indicate the best performance among the compared methods (MWGB-Cox model).

In the independent internal validation cohort, 7 patients (28.0%) had died and 18 patients (72.0%) had confirmed 1-year survival status at the 1-year landmark. No patient was censored before the 1-year landmark, ensuring reliable estimation of the primary endpoint. To evaluate the clinical utility of the prognostic models, Kaplan-Meier survival curves stratified by risk group were performed (**Figure 5**). To facilitate clinical implementation, a prognostic nomogram based on the MWGB-Cox model was constructed (**Figure 6A**). This enables clinicians to estimate individual prognosis quantitatively and inform shared decision-making. Furthermore, decision curve analysis (DCA) was performed on the MWGB-Cox model to assess net benefit for 1-year survival prediction. Decision curve analysis in the independent internal validation cohort demonstrated that the MWGB-Cox model provided positive and superior net benefit compared with both treat-all and treat-none strategies across threshold probabilities of 0.10–0.60 (**Supplementary Table S3**; **Figure 6B**).

**Figure 5:**
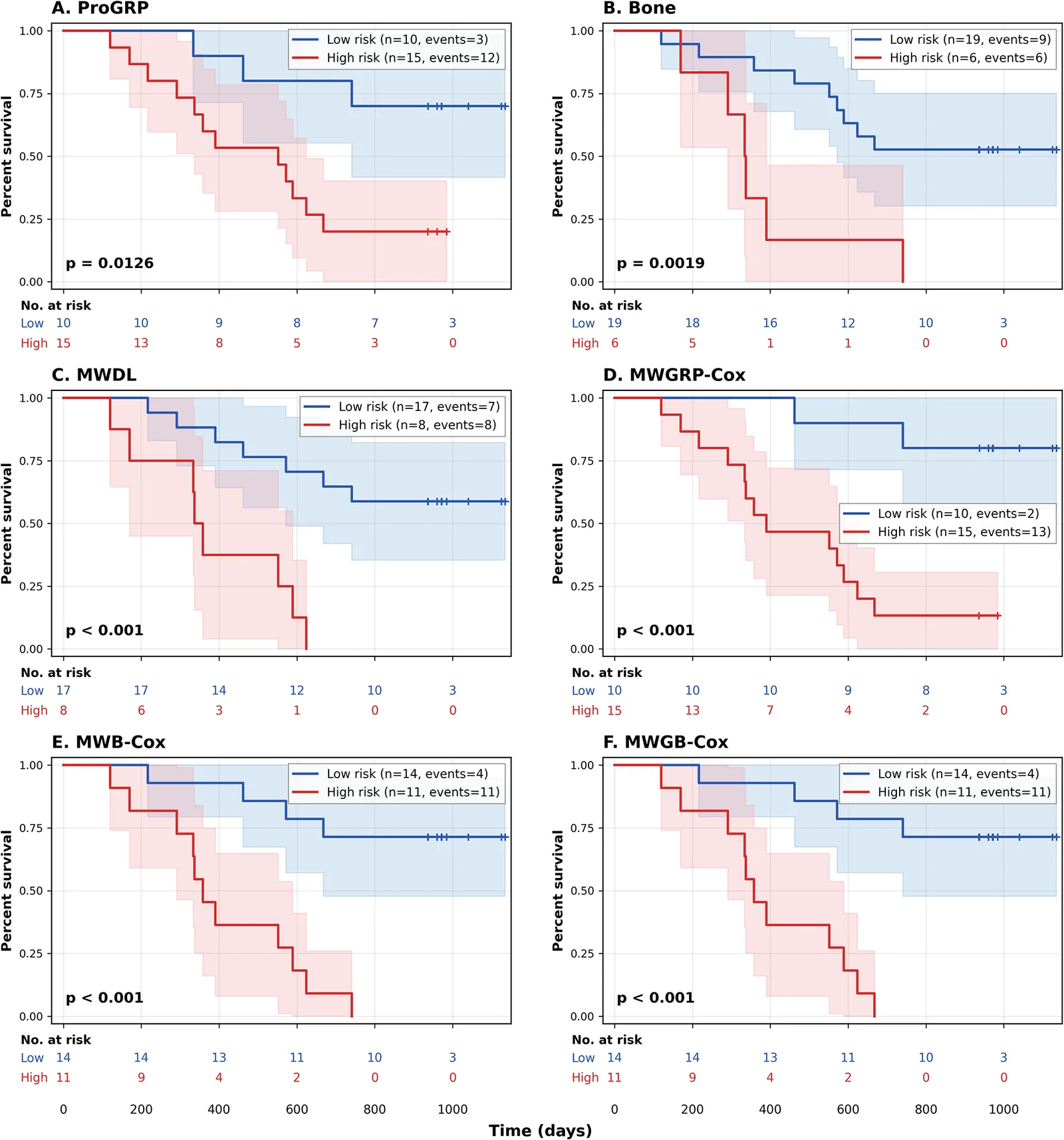
Kaplan-Meier survival curves stratified by risk groups in the independent internal validation set. Patients were classified into high-risk (red) and low-risk (blue) groups based on predictions from six different methods: **(A)** ProGRP, **(B)** bone metastasis, **(C)** MWDL model, **(D)** MWGRP-Cox model, **(E)** MWB-Cox model, and **(F)** MWGB-Cox model.

**Figure 6:**
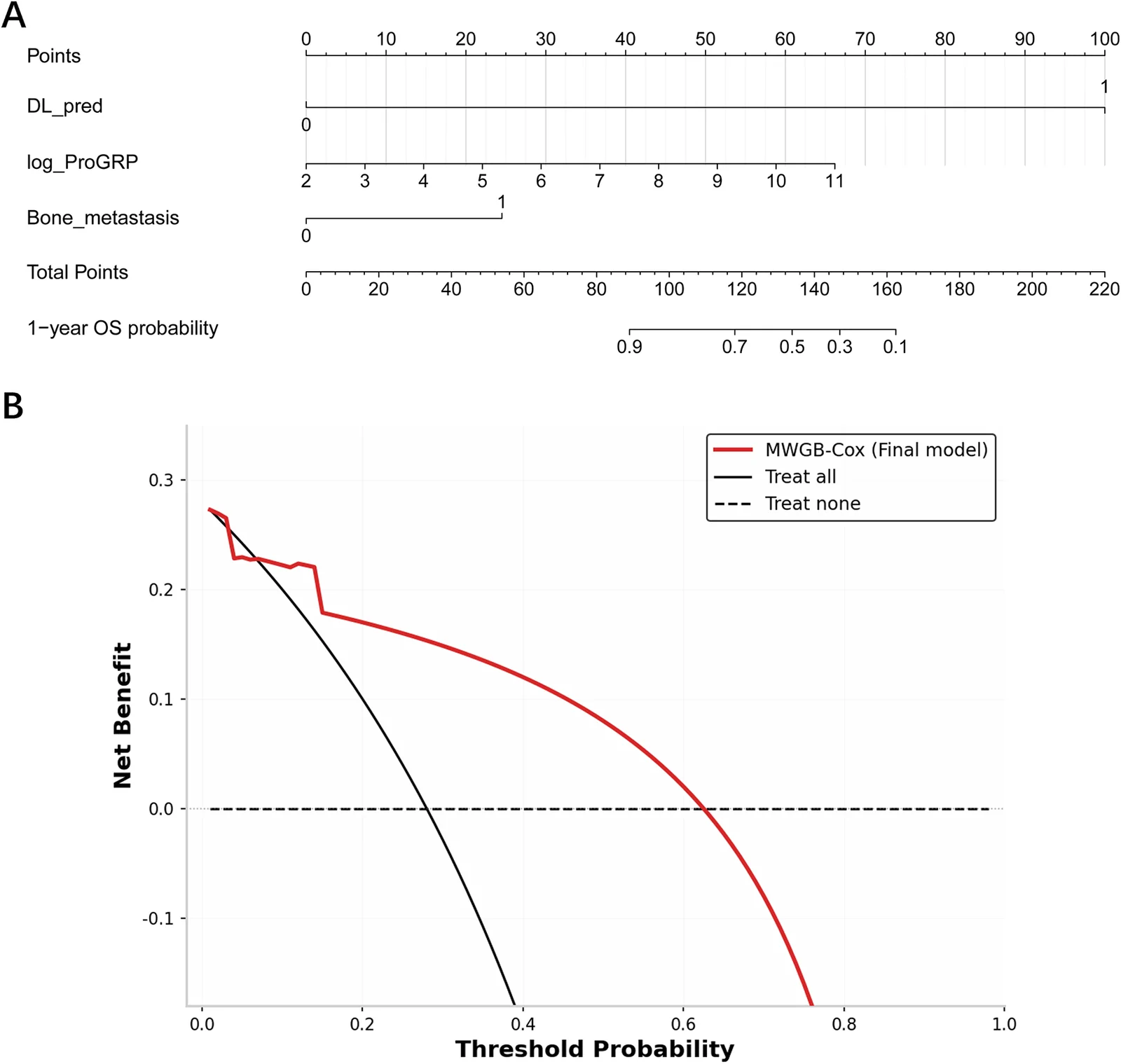
Clinical utility of the MWGB-Cox model. **(A)** Prognostic nomogram for predicting 1-year overall survival. To use the nomogram, locate the patient's DL-predicted risk (0 or 1), log(ProGRP + 1) value, and bone metastasis status on the respective axes, draw a vertical line upward to the "Points" axis to determine the score for each variable, sum the points, and locate the total on the "Total Points" axis to obtain the predicted 1-year survival probability. **(B)** Decision curve analysis in the independent internal validation cohort comparing the net benefit of the MWGB-Cox model against treat-all and treat-none strategies.

## Discussion

Therapeutic options for ES-SCLC remain limited, with only 14% of patients deriving additional benefit from ICIs. Furthermore, fatal immune-related adverse events, such as immune-related pneumonitis and myocarditis, are not uncommon. Therefore, the establishment of efficient and accurate predictive models for immunotherapeutic response and the accurate identification of patient subgroups that benefit from immunotherapy have become urgent clinical priorities in the management of ES-SCLC. In the present study, we analyzed the clinical characteristics and laboratory investigations in 99 ES-SCLC patients, and are the first to develop a novel model based on ProGRP, bone metastases and CT-based DL. This novel MWGB-Cox model enables rapid and cost-effective prediction of immunotherapy outcomes in ES-SCLC patients who received immunotherapy as first-line treatment.

A number of biomarkers, such as TMB, PD-L1 expression, and GRIm-Score, have been proposed to assess the efficacy of ICIs and predict survival outcomes in patients with SCLC (6, 7). However, the predictive performance of these biomarkers has been unsatisfactory, as reflected by their low AUC values. Recently, deep learning has emerged as a powerful tool for medical image analysis, offering distinct advantages over conventional radiomics approaches. Unlike radiomics, which relies on predefined handcrafted feature engineering, such as shape, texture, and wavelet features, constrained by mathematical formulations, deep learning models automatically extract hierarchical feature representations directly from imaging data in an end-to-end manner, potentially capturing subtle and complex patterns that may not be accessible through manual feature design (22). Long et al. conducted a multicenter study comparing deep learning models with radiomics models for ternary classification of pulmonary ground-glass nodule invasiveness, demonstrating that deep learning achieved superior performance (23). Furthermore, systematic reviews have demonstrated that fusion models combining deep learning and radiomics generally outperform unimodal approaches across various cancer types (24, 25). Consistent with these findings, our study determined that deep learning models developed from CT images of the lung window (LWDL) or mediastinum window (MWDL) exhibited superiority over the ProGRP and bone metastasis in predicting 1-year OS of ES-SCLC patients receiving first-line immunotherapy. This highlights the potential of CT-driven AI technologies, while also indicating room for improvement of AUC and sensitivity. The multimodal MWGB-Cox model, constructed by integrating MWDL with log(ProGRP + 1) levels and bone metastasis, not only achieved a substantially higher AUC for 1-year OS prediction than the MWDL model, but also effectively overcame the limitation of insufficient sensitivity. These results support the emerging consensus that deep learning-based and multimodal approaches hold substantial promise for non-invasive prediction of immunotherapy outcomes, potentially facilitating personalized treatment decision-making in clinical practice. In the independent internal validation cohort, stepwise improvements from MWDL (AUC = 0.774) to MWGRP-Cox (AUC = 0.817) to MWB-Cox (AUC = 0.853) to MWGB-Cox (AUC = 0.873) exhibited prognostic performance comparable to the machine learning model of pathological images reported by Shibaki et al. (26), while our novel MWGB-Cox model was more simpler, more cost-effective, and more suitable for widespread clinical application.

In the present study, the median OS of the training set patients was 16.9 months, which is higher than the median OS reported in the IMpower133 trial (4) and the CASPIAN trial (5). Several factors may contribute to this outcome. Firstly, our cohort exclusively included patients who received at least two cycles of chemotherapy combined with immunotherapy at the beginning of the treatment, and completed all treatments at our hospitals. Patients who were lost to follow-up after one cycle due to poor response/adverse reaction, or who were referred to other hospitals, were excluded. As a result, the enrolled patients had better socioeconomic status and higher treatment adherence. Secondly, for DL-based image analysis, we utilized baseline images obtained at initial diagnosis. Therefore, patients with extensive atelectasis or massive pleural effusion that not easy to accurate delineate of the primary lesion were excluded, which may have led to the exclusion of patients with high tumor burden. Thirdly, a large proportion of our patients received radiotherapy. What’s more, some patients even underwent radiotherapy at multiple sites or multiple courses at the same site, including thoracic radiotherapy, cranial radiotherapy, and radiotherapy for bone metastatic lesions. This is consistent with recent studies demonstrating that sequential thoracic radiotherapy following chemoimmunotherapy could greatly improve OS in ES-SCLC patients (27, 28). In addition, several patients received local therapies for metastatic sites, such as ablation therapy for liver metastases. Collectively, these factors may explain the favorable OS observed in our study population.

ES-SCLC is characterized by its high malignancy and extensive tumor cell infiltration, which contribute to impaired immune surveillance and reduced tumor-killing activity. Nevertheless, the precise mechanisms driving tumor immune evasion remain poorly understood. Gastrin-releasing peptide (GRP), a key member of the mammalian bombesin-like peptide family, binds to the GRP receptor (GRPR) on SCLC cell surfaces, forming an autocrine loop that facilitates tumor proliferation (29). Previous studies have demonstrated that there was a significant negative correlation between ProGRP levels and T lymphocytes, alongside a positive association between ProGRP levels and regulatory T cells (Tregs) (8). These findings demonstrated that ProGRP played a pivotal role in immune evasion mechanisms. Consistent with this evidence, our study revealed significantly elevated ProGRP levels in patients with OS <1 year compared to those with OS ≥1 year. However, further investigations are required to elucidate ProGRP’s role in modulating T lymphocyte-mediated tumor-killing activity and the molecular mechanisms underlying this process.

Bone metastasis, another factor identified in this study, exerts a significant impact on the efficacy of immunotherapy in ES-SCLC patients. Multivariate COX regression analysis revealed that bone metastasis had a more pronounced impact on patient prognosis than either liver or brain metastases. Previous studies in other tumor types such as NSCLC have demonstrated that the presence of baseline bone metastasis has a limited impact on chemotherapy outcomes, but it significantly reduced the OS in patients receiving immunotherapy (30). Even with dual immunotherapy regimens, the negative impact of bone metastasis on treatment efficacy could not be reversed (31). Cheng JN, et al. reported that bone metastasis induced excessive secretion and activation of osteopontin, which suppresses CD8+ T-cell activity, resulting in an immunosuppressive tumor microenvironment characterized as “cold tumor” phenotype and consequently diminishing responsiveness to immunotherapy (32). Therefore, therapeutic agents targeting GRP and/or osteopontin may hold promise for restoring the sensitivity of ES-SCLC patients to immunotherapy in the future.

The recognition of immunotherapy’s hallmark characteristics, delayed therapeutic onset and long-tailed survival effects, has led to growing awareness among oncologists that conventional evaluation metrics such as RECIST criteria and progression-free survival (PFS) inadequately reflect long-term clinical benefits of immunotherapy. Furthermore, mounting clinical evidence demonstrates that PFS improvements frequently fail to translate into OS advantages (33, 34). This paradigm shift has prompted the U.S. Food and Drug Administration (FDA) to retract multiple accelerated approvals initially granted based on objective response rate or PFS data from clinical trials (35). Informed by these evolving evidentiary standards, our study employs 1-year OS, which is currently identified as the only validated surrogate endpoint for cancer immunotherapy (20), to develop the novel predictive model in ES-SCLC patients who received with ICIs as first-line treatment.

However, this study also has several limitations. First, although our study cohort was not smaller than those reported in other ES-SCLC studies (36), and was even larger than some multicenter investigations (26, 27), the relatively modest sample size may limit statistical power and generalisability. What’s more, the validation cohort were recruited from an independent internal validation from a different division of the same institution. Despite the Nansha Division being a legally independent entity, it belongs to the same affiliated hospital network as the training cohort and shares standardized imaging protocols and clinical training pathways. Patients from both divisions share similar imaging protocols, treatment guidelines, and case mix, which may limit the generalizability of our findings to other centers with different practice patterns. In the present study, external multi-center validation was not performed due to the limited research budget. Thus, before applying this model to guide immunotherapy decision-making for ES-SCLC patients, larger-scale prospective multicenter clinical trials are needed to further validate its predictive performance. Second, the possibility of the background of the ES-SCLC primary lesion CT image affecting the model performance cannot be ruled out. Third, the molecular mechanisms involved in immune evasion mediated by ProGRP and bone metastasis in ES-SCLC patients remain to be elucidated in the future.

## Conclusions

In summary, we developed a novel multimodal prognostic model (MWGB-Cox) by integrating CT-derived deep learning features, serum log(ProGRP + 1) levels, and bone metastasis status. This model enables rapid, cost-effective prediction of 1-year OS in ES-SCLC patients receiving first-line immunotherapy. The proposed MWGB-Cox model demonstrates robust clinical utility, offering actionable insights for optimizing therapeutic decision-making, minimizing unnecessary treatment expenditures, and stratifying patient subgroup most likely to benefit from immune-based therapies.

## Data Availability

The original contributions presented in the study are included in the article/**Supplementary Material**. Further inquiries can be directed to the corresponding authors.
