# Supplementary material for: A novel multimodal model integrating CT-based deep learning and clinical information for predicting 1-year OS of first-line immunotherapy in ES-SCLC patients
Source: Front Oncol. 2026 Sep 30;16:1901931. doi: 10.3389/fonc.2026.1901931 (PMC13661403; doi:10.3389/fonc.2026.1901931)
Supplement: Supplementary file 1 [file SupplementaryFile1.docx]

**Supplementary Note.** Detailed model development and validation metrics are summarized in **Supplementary Table S1**, including sample size, events per variable (EPV = 17.0), bootstrap optimism-corrected C-index (0.830), bootstrap optimism (0.0056), Z statistic (3.32), p-value (0.0009), variable selection frequencies, calibration slope (0.930), integrated Brier score (IBS = 0.030), and the proportion of patients with predicted 1-year survival probability >0.90 (81.8%). **Supplementary Figure S1** shows the distribution of predicted 1-year survival probabilities in the training cohort. **Supplementary Figure S4** presents bootstrap-corrected calibration curves (B = 300) for 1-year and 2-year overall survival. **Supplementary Figure S5** illustrates the bootstrap variable selection stability (B = 500).

**Table S1. Model Development and Validation Details**

| **Category** | **Item** | **Value** |
| --- | --- | --- |
| Sample size | Training cohort, n | 99 |
|  | Validation cohort, n | 25 |
|  | Training cohort events (overall mortality), n | 51 |
|  | Validation cohort events (overall mortality), n | 15 |
|  | Training cohort 1-year mortality, n (%) | 22 (22.2%) |
|  | Validation cohort 1-year mortality, n (%) | 7 (28.0%) |
|  | Events per variable (EPV) | 17 |
| Bootstrap validation | Resamples, B | 500 |
|  | Optimism-corrected C-index | 0.83 |
|  | Bootstrap optimism | 0.0056 |
|  | Z statistic | 3.32 |
|  | p-value | 0.0009 |
| Variable selection frequency | DL_pred | 95.4% |
|  | log_ProGRP | 89.8% |
|  | Bone_metastasis | 94.2% |
| Calibration | Calibration slope | 0.93 |
|  | Integrated Brier Score (IBS) | 0.03 |
|  | Predicted 1-year survival probability >0.90, % | 81.8% |

**Table S2. Subgroup Analysis of MWGB-Cox Model Performance**

| **Subgroup** | **N** | **Events** | **C_index** | **C_index_95%CI** | **HR** | **HR_95CI** | **p_value** |
| --- | --- | --- | --- | --- | --- | --- | --- |
| Overall | 99 | 51 | 0.836 | 0.789–0.883 | 2.72 | 2.24–4.06 | <0.001 |
| DL_pred |  |  |  |  |  |  |  |
| 0 | 81 | 33 | 0.730 | 0.661–0.799 | 3.18 | 1.98–6.33 | <0.001 |
| 1 | 18 | 18 | 0.722 | 0.587–0.857 | 2.41 | 1.26–11.19 | 0.113 |
| Bone metastasis | |  |  |  |  |  |  |
| No | 64 | 22 | 0.773 | 0.703–0.843 | 3.40 | 2.57–6.21 | <0.001 |
| Yes | 35 | 29 | 0.844 | 0.773–0.915 | 2.27 | 1.69–5.06 | 0.003 |
| ProGRP |  |  |  |  |  |  |  |
| ≤ median | 50 | 19 | 0.821 | 0.751–0.891 | 3.06 | 2.54–5.58 | <0.001 |
| > median | 49 | 32 | 0.794 | 0.724–0.864 | 2.54 | 1.91–5.85 | 0.001 |
| Interaction: DL_pred × Bone metastasis | — | — | — | — | — | — | 0.163 |
| Note: HR represents the hazard ratio per 1-SD increase in the MWGB-Cox linear predictor. C-index 95% CI was estimated by 500 bootstrap resamples. | | | | | | | |

**Table S3. Net benefit comparison of the MWGB-Cox model, treat-all, and treat-none strategies across clinically relevant threshold probabilities in the independent internal validation cohort (n = 25).**

| Threshold probability | MWGB-Cox  (Model) | Treat-all | Treat-none | Net gain  (Model vs. treat-all) |
| --- | --- | --- | --- | --- |
| 0.1 | 0.222 | 0.2 | 0 | 0.022 |
| 0.2 | 0.17 | 0.1 | 0 | 0.07 |
| 0.3 | 0.149 | −0.029 | 0 | 0.177 |
| 0.4 | 0.12 | −0.200 | 0 | 0.32 |
| 0.5 | 0.08 | −0.440 | 0 | 0.52 |
| 0.6 | 0.02 | −0.800 | 0 | 0.82 |
| 0.7 | −0.080 | −1.400 | 0 | 1.32 |

**Figure S1.** Distribution of predicted 1-year survival probabilities in the training cohort (n = 99). The dashed vertical line indicates the predicted probability threshold of 0.90.

**Figure S2.** Schoenfeld residual plots for the MWGB-Cox model. (A) DL_pred; (B) log(ProGRP + 1); (C) Bone_metastasis. The solid line represents the smoothed residual trend; the dashed lines represent the 95% confidence band.

**Figure S3.** Restricted cubic spline (4 knots) analysis of the relationship between ProGRP and overall survival in the training cohort. The solid line represents the estimated log hazard ratio; the shaded area represents the 95% confidence interval. The reference value is the median ProGRP.

**Figure S4.** Bootstrap-corrected calibration curves (B = 300) for the MWGB-Cox model in the training cohort. The dashed diagonal line represents perfect calibration; the solid line represents the bias-corrected calibration curve; the dotted line represents the apparent calibration; the rug plot at the top indicates the distribution of predicted probabilities.

**Figure S5.** Bootstrap variable selection stability (B = 500). The bar chart shows the frequency (%) with which each variable achieved statistical significance (p < 0.05) in multivariate Cox models fitted to bootstrap resamples.
